# Supplementary material for: WordCommentsAnalyzer: A windows software tool for qualitative research
Source: F1000Res. 2018 Sep 4;7:536. Originally published 2018 May 3. [Version 2] doi: 10.12688/f1000research.14819.2 (PMC6137411; doi:10.12688/f1000research.14819.2)
Supplement: Supplementary file 3 [file f1000research-7-17614-s0002.tgz › b2a79496-4ada-47c4-9682-a99ce867836d.zip!/Eight Iranian Health Journals/Journal of Education and Community Health_1.docx]

© Journal of Education and Community Health. This work is licensed under CC BY-NC 4.0 (https://creativecommons.org/licenses/by-nc/4.0/).

Volume & Issue: دوره 4 - شماره 4

Title: تأثیر آموزش مبتنی بر مدل اعتقاد بهداشتی در پیشگیری از بیماریهای مقاربتی در زوجین شرکت کننده در کلاس های آموزشی پیش از ازدواج

http://jech.umsha.ac.ir/article-1-412-fa.html

سابقه و هدف: بیماریهای مقاربتی بعنوان یکی از شایعترین بیماریهای عفونی از معضلات بهداشتی جامعه می باشد. اجرای مداخلات آموزش بهداشت بهترین استراتژی در دسترس برای ارتقای بهداشت جنسی محسوب می شود.  لذا مطالعه حاضر با هدف بررسی تأثیر آموزش پیشگیری از بیماریهای مقاربتی در زوجین بر اساس مدل اعتقاد بهداشتی در  شهر فسا  در سال 1395  انجام گرفت. مواد و روش ها: این مطالعه نیمه تجربی برروی 70 زوج مراجعه کننده به مراکز بهداشتی درمانی شهر فسا (35 زوج در گروه مداخله و 35زوج در گروه کنترل) انجام گرفت. اﺑﺰار ﮔﺮدآوری اﻃﻼﻋﺎت، پرسشنامه ای استاندارد شامل اطلاعات دموگرافیک، سنجش سازه های مدل اعتقاد بهداشتی و عملکرد زوجین در پیشگیری از بیماری های مقاربتی بود که توسط هر دو گروه قبل و 3 ماه بعد از مداخله تکمیل شد. مداخله آموزشی طی 4 جلسه 50-55 دقیقه ای به شیوه بحث گروهی، پرسش و پاسخ همراه با تصاویر آموزشی برای گروه مداخله اجرا شد. داده ها وارد نسخه 22 نرم افزار SPSS و با استفاده از آزمون های آماری مجذور کای، تی مستقل و تی زوجی تحلیل گردید. یافته ها: میانگین سنی افراد شرکت کننده در گروه مداخله و کنترل به ترتیب 5.35±27.02 و 4.45±25.14 بود. میانگین نمرات تمام سازه های مدل اعتقاد بهداشتی و میزان عملکرد زوجین در پیشگیری از بیماری های مقاربتی در گروه مداخله نسبت به گروه کنترل بعد از مداخله ی آموزشی افزایش یافت (0.001>P). نتیجه گیری: نتایج این مطالعه نشان داد که طراحی و اجرای برنامه های آموزشی مبتنی بر مدل اعتقاد بهداشتی در زمینه پیشگیری از بیماریهای مقاربتی و بهداشت جنسی اثربخش است.

Title: بررسی ارتباط سواد سلامت و کیفیت زندگی در بزرگسالان شهر کرج

http://jech.umsha.ac.ir/article-1-403-fa.html

سابقه و هدف: سواد سلامت به عنوان یکی از موضوعات مهم برای ارتقای سطح سلامت جامعه، مورد توجه سیاستگزاران قرار گرفته است. از طرفی کیفیت زندگی و تلاش در راه اعتلای آن، نقش بسزایی در سلامت فردی و اجتماعی دارد. نظر به نقش بزرگسالان به عنوان گروه مولد جامعه، این پژوهش با هدف تعیین ارتباط سواد سلامت و کیفیت زندگی در بزرگسالان انجام شد.   مواد و روش ها: این مطالعه مقطعی از نوع همبستگی بر روی 256 نفر از بزرگسالان 18 تا 65 ساله شهر کرج در سال 95 انجام شد. نمونه گیری به صورت نمونه گیری در دسترس انجام شد. داده‌ها با استفاده از ابزار سنجش سواد سلامت جمعیت شهری 18 تا 65 ساله ایرانی (HELIA) و پرسشنامه کیفیت زندگی 12 سوالی (SF-12) گردآوری شدند. سپس با استفاده از آماره‌های توصیفی و آزمون‌ همبستگی پیرسون در نرم افزار spss نسخه 21 تحلیل شدند. یافته ها: در این مطالعه 24.2 درصد (62 نفر) از بزرگسالان، سواد سلامت ناکافی و 23.4 درصد (60 نفر) سواد سلامت نه چندان کافی داشتند. میانگین (انحراف معیار) نمره کیفیت زندگی در حیطه های سلامت جسمانی (2.68)16.24، سلامت روانی (3.89)20.43 و کیفیت زندگی کل (5.82)36.68 از 48 بود. بین سواد سلامت و ابعاد جسمانی و روانی کیفیت زندگی و همچنین کیفیت زندگی کل همبستگی مستقیم و  معنی دار وجود داشت (0.05>P). نتیجه گیری: با توجه به ارتباط سواد سلامت با ابعاد کیفیت زندگی، توجه هر چه بیشتر به ارتقای سواد سلامت افراد (به ویژه بزرگسالان دارای سواد سلامت ناکافی) در برنامه های ارتقای کیفیت زندگی، ضروری به نظر می رسد.

Title: رفتارهای غربالگری سرطان پروستات و باورهای مرتبط با آن در بین مردان 50 تا 70 ساله شهر همدان: ارزیابی تهدید و مقابله

http://jech.umsha.ac.ir/article-1-344-fa.html

سابقه و هدف: سرطان پروستات یکی از بیماری های شایع در مردان می‌باشد و انجام رفتار غربالگری نقش مهمی در کاهش موارد سرطان پروستات ایفا می‌کند. مطالعه حاضر با هدف تحلیل باورهای مرتبط با رفتارهای غربالگری سرطان‌پروستات دربین مردان مراجعه کننده به مراکز بازنشستگی شهر همدان انجام گردید. مواد و روش‌ها: این مطالعه مقطعی در سال 1395 بر روی 403 نفر از مردان مراجعه کننده به کانون‌های بازنشستگی شهر همدان که با روش نمونه گیری تصادفی طبقه ای انتخاب شده بودند، انجام شد. ابزار گردآوری اطلاعات شامل پرسشنامه­ ای مشتمل بر متغیرهای جمعیت شناختی و سازه های تئوری انگیزش محافظت بود. اطلاعات در نرم­افزار آماری  SPSS-16 و با بهره­گیری از آماره های توصیفی و آزمون همبستگی پیرسون تحلیل گردید. یافته ها: میانگین سن مردان شرکت کننده در این مطالعه 5.74±60.2 سال بود. طبق یافته ها میزان انجام آزمایش اختصاصی پروستات (PSA) و معاینه انگشتی رکتوم (DRE) به ترتیب 21.6 و 5.7 درصد گزارش شد که در سطح نامطلوبی قرار داشتند. نتایج مطالعه نشان داد که وضعیت حساسیت درک شده نسبت به سرطان پروستات با 44.91 درصد در سطح نامطلوبی قرار داد، ولیکن وضعیت کارایی پاسخ درک شده و پاداش درک شده با 77.08 و 75.9 در حد نسبتاً مطلوبی می باشد. نتیجه‌گیری: بر اساس یافته های مطالعه حساسیت درک شده، پاداش درک شده، ترس و خودکارآمدی درک شده نامطلوب بوده اند که در برنامه ریزی مداخلات آموزشی توجه به این یافته ها ضروری به نظر می رسد.

Title: اثربخشی مداخله تلفیقی شناختی رفتاری و پذیرش و تعهد بر اختلال اضطراب فراگیر در بین دانشجویان

http://jech.umsha.ac.ir/article-1-395-fa.html

سابقه و هدف: با توجه به اهمیت اختلال اضطراب فراگیر، مطالعه حاضر با هدف تعیین تاثیر مداخله تلفیقی شناختی- رفتاری و پذیرش و تعهد بر اختلال اضطراب فراگیر در بین دانشجویان دانشگاه آزاد اسلامی شهر تویسرکان انجام شد. مواد و روش‌ها: پژوهش حاضر یک مطالعه مداخله ای از نوع پیش آزمون و پس آزمون بود که در سال 1396 انجام شد. 18 نفر از دانشجویان در دسترس که برای دریافت مشاوره به مرکز مشاوره دانشگاه آزاد تویسرکان مراجعه کرده بودند با تشخیص ملاک های DSM5 توسط روانپزشک، انتخاب و وارد مطالعه شدند. ابزار گردآوری مطالعه شامل پرسشنامه دو بخشی اطلاعات دموگرافیک و پرسشنامه استاندارد بک بود. برنامه آموزشی تلفیقی شامل 12 جلسه آموزشی بود. داده های به دست آمده از مطالعه با استفاده از نرم افزار آماری SPSS نسخه 16 و همچنین با استفاده از آزمون تی زوجی و کای دو تجزیه و تحلیل شدند. یافته ها: نتایج حاصل از این مطالعه نشان داد مداخله تلفیقی باعث کاهش میانگین اضطراب فراگیر به مقدار 8.27 در بین دانشجویان شده است. همچنین مداخله تلفیقی باعث شد 83.3 درصد از دانشجویانی که دارای اختلال اضطراب فراگیر شدید بودند، بعد از انجام مداخله اضطراب آنان کمتر شده و در درجه اضطراب فراگیر متوسط قرار گیرند. نتیجه گیری: نتایج پژوهش نشان داد که مداخله تلفیقی یک مداخله اثربخش در کاهش علایم اختلال اضطراب فراگیر است و این مداخله همانند مداخله شناختی– رفتاری و مداخله پذیرش و تعهد موجب کاهش علایم اختلال اضطراب فراگیر در مبتلایان می شود.

Title: سبک زندگی ساکنین جزیره هرمز: یک مطالعه مقطعی

http://jech.umsha.ac.ir/article-1-379-fa.html

سابقه و هدف: بیماری‌های متأثر از سبک زندگی از عمده‌ترین علل مرگ و میر در ایران می‌باشند. شواهد موجود حاکی از وجود ارتباط بین سبک زندگی ناسالم با بروز بیماری‌ها و مشکلات سلامتی هستند. در این راستا، مطالعه حاضر با هدف تعیین وضعیت سبک زندگی و عوامل مرتبط با آن در بین ساکنین جزیره هرمز انجام شد. مواد و روش‌‌ها: در این مطالعه مقطعی 400 نفر از ساکنین بالای 15 سال شهر هرمز به‌صورت نمونه‌گیری خوشه‌ای دو مرحله‌ای در سال 1395 مورد مطالعه قرار گرفتند. در این پژوهش اطلاعات جمعیت‌شناختی و عوامل مربوط به سبک زندگی با استفاده از پرسشنامه محقق‌ساخته جمع‌آوری گردیدند. داده‌ها نیز با استفاده از نرم‌افزار آماری SPSS 19 و با به‌کارگیری آماره‌های توصیفی و آزمون‌های کای‌دو و آنالیز واریانس یک‌طرفه تحلیل شدند. یافته‌ها: میانگین و انحراف معیار سنی افراد مورد مطالعه 14.52±36.1 سال بود. از نظر سبک زندگی میزان مصرف روغن مایع، غذای کم‌نمک، فعالیت فیزیکی منظم و عدم مصرف دخانیات به‌ترتیب 81، 20.3، 37 و 68.3 درصد برآورد شد. میانگین مصرف میوه، سبزیجات و ماهی نیز به‌ترتیب 2.48، 3.55 و 3.65 واحد غذایی در طول هفته گزارش گردید. همچنین ارتباط آماری معناداری بین سطح تحصیلات و مصرف نمک (0.002=P)، وضعیت اقتصادی و نوع روغن مصرفی (0.005>P) و الگوی غذایی سالم با وضعیت اقتصادی افراد (0.05>P) مشاهده شد. نتیجه‌گیری: مطابق با یافته‌ها، ارتقای سطح آگاهی و دانش تغذیه‌ای، آگاهی از مضرات استعمال دخانیات در افراد با تحصیلات پایین، پیاده‌سازی سیاست‌های ارزان و دردسترس‌بودن مواد غذایی سالم، افزایش مالیات دخانیات، فراهم‌نمودن مقدمات فعالیت فیزیکی، اطلاع‌رسانی و هنجارسازی در زمینه رفتارهای ایمنی جهت ارتقای سبک زندگی اهمیت به‌سزایی دارد.

Title: پیش‌بینی نارضایتی از تصویر بدن بر پایه‌ی هوش معنوی و الگوهای فرهنگی ـ اجتماعی در زنان مراجعه کننده به باشگاه‌های ورزشی شهر یزد

http://jech.umsha.ac.ir/article-1-366-fa.html

سابقه و هدف: مشغولیت با تصویر بدنی از مهمترین موارد د‌ل‌مشغولی افراد بوده که عوامل زیادی بر میزان رضایت از آن تاثیر می­ گذارند. هدف از پژوهش حاضر پیش‌بینی نارضایتی از تصویر بدن بر پایه‌ی هوش معنوی و الگوهای فرهنگی­ اجتماعی در زنان مراجعه‌کننده به باشگاه‌های ورزشی شهر یزد بود. مواد و روش ها: پژوهش حاضر توصیفی از نوع همبستگی می ­باشد. جامعه‌ی آماری مورد مطالعه را کلیه زنان ساکن شهر یزد تشکیل می‌داد که در کلاس­های ورزشی شرکت می­ کردند. با استفاده از روش نمونه‌گیری تصادفی خوشه‌ای تعداد 351 نفر جهت شرکت در پژوهش انتخاب شدند. ابزار پژوهش شامل پرسشنامه­ های تصویر بدن، هوش معنوی و الگوهای فرهنگی­ اجتماعی بود. اطلاعات جمع­ آوری شده با استفاده از نرم افزار SPSS و آمار توصیفی (میانگین و انحراف معیار) و آمار استنباطی شامل ضریب همبستگی پیرسون و تحلیل رگرسیون چند متغیره مورد بررسی قرار گرفت. یافته ها: نتایج نشان داد بین هوش معنوی و نارضایتی از تصویر بدن همبستگی معکوس و بین الگوهای فرهنگی-اجتماعی و نارضایتی از تصویر بدن همبستگی معنادار مثبت وجود داشت (P<0.05). هوش معنوی و الگوهای فرهنگی-اجتماعی هر دو پیش بینی کننده نارضایتی از تصویر بدن هستند اما هوش معنوی سهم بیشتری در تبیین آن دارد. نتیجه‌گیری: در مجموع این پژوهش نشان داد که متغیرهای هوش معنوی و الگوهای اجتماعی-فرهنگی نقش معناداری در تبیین نارضایتی از تصویر بدنی  دارند اما هوش معنوی پیش‌بینی‌کننده قوی‌تری برای نارضایتی از تصویر بدنی بود. از این رو بررسی اثربخشی جلسات آموزش هوش معنوی جهت کاهش نارضایتی از تصویر بدنی برای پژوهش های آتی پیشنهاد می‌گردد.

Title: رابطه‌ی بین حمایت اجتماعی ادراک‌شده و خودشکوفایی با امید به زندگی در سالمندان شهر تهران

http://jech.umsha.ac.ir/article-1-378-fa.html

سابقه و هدف: با توجه به جمعیت رو به رشد سالمندان ایران، بررسی امید به زندگی و عوامل مرتبط با آن ضروری می‌باشد؛ از این رو پژوهش حاضر با هدف تعیین رابطه‌ بین حمایت اجتماعی ادراک‌شده و خودشکوفایی با امید به زندگی در سالمندان شهر تهران انجام شد. مواد و روش‌‌ها: این مطالعه توصیفی- مقطعی در سال 1395 در ارتباط با 200 نفر از سالمندان شهر تهران (سالمندان مقیم سراهای سالمندان و سالمندان غیرمقیم در این مراکز) انجام شد. روش نمونه‌گیری به‌صورت در دسترس بود. به‌منظور جمع‌آوری داده‌ها از پرسشنامه‌های‌ خودشکوفایی Maslow، امید به زندگی Miller و حمایت اجتماعی ادراک‌شده‌ Norbeck استفاده شد. داده‌ها توسط نرم‌افزار SPSS 20 و با استفاده از آزمون‌های همبستگی Pearson و تحلیل رگرسیون تجزیه ‌و تحلیل گردیدند. یافته‌ها: نتایج نشان داد که بین حمایت اجتماعی ادراک‌شده و امید به زندگی، خودشکوفایی و امید به زندگی و خودشکوفایی و حمایت اجتماعی ادراک‌شده‌ سالمندان شهر تهران رابطه‌ مثبت و معناداری وجود دارد (0.05>P). نتایج تجزیه ‌و تحلیل رگرسیون نیز بیانگر آن بود که متغیرهای خودشکوفایی و حمایت اجتماعی تأثیر مستقیمی بر امید به زندگی دارند. بر مبنای نتایج، حمایت اجتماعی ادراک‌شده در سالمندان مقیم سراهای سالمندان و خودشکوفایی در سالمندان غیرمقیم در این مراکز تأثیر بیشتری بر امید به زندگی ‌آن‌ها داشته‌اند (0.05>P). نتیجه‌گیری: یافته‌های مطالعه حاضر حاکی از آن است که با فراهم‌آوردن زمینه‌های مناسب جهت بهبود کیفیت، تقویت و ایجاد شاخصه‌های حمایت اجتماعی ادراک‌شده و خودشکوفایی در سالمندان (سالمندان مقیم سراهای سالمندان و سالمندان غیرمقیم در این مراکز) ‌می‌توان امید به زندگی را در ‌آن‌ها بهبود بخشید.
